# Pathogenicity of GI-23 Avian Infectious Bronchitis Virus Strain Isolated in Brazil

**DOI:** 10.3390/v15051200

**Published:** 2023-05-19

**Authors:** Iara Maria Trevisol, Luizinho Caron, Marcos Antônio Zanella Mores, Daiane Voss-Rech, Gabriel da Silva Zani, Alberto Back, Jorge Augusto Petroli Marchesi, Paulo Augusto Esteves

**Affiliations:** 1 Embrapa Suínos e Aves, Concordia 89715-899, SC, Brazil; luizinho.caron@embrapa.br (L.C.); marcos.mores@embrapa.br (M.A.Z.M.); daiane.rech@embrapa.br (D.V.-R.);; 2 Department of Veterinary Preventive, Faculty of Veterinary, Federal University of Pelotas, Pelotas 96010-900, RS, Brazil; 3 MercoLab Laboratórios, Cascavel 85816-280, PR, Brazil

**Keywords:** IBV variant, Brazil GI-23 isolate, chicken disease, South America, S1 complete sequence

## Abstract

IBV variants belonging to the GI-23 lineage have circulated since 1998 in the Middle East and have spread to several countries over time. In Brazil, the first report of GI-23 occurred in 2022. The study aimed to evaluate the in vivo pathogenicity of exotic variant GI-23 isolates. Biological samples were screening by real-time RT-PCR and classified in to GI-1 or G1-11 lineages. Interestingly, 47.77% were not classified in these lineages. Nine of the unclassified strains were sequenced and showed a high similarity to the GI-23 strain. All nine were isolated and three, were studied for pathogenicity. At necropsy, the main observations were the presence of mucus in the trachea and congestion in the tracheal mucosa. In addition, lesions on the tracheas showed marked ciliostasis, and the ciliary activity confirmed the high pathogenicity of isolates. This variant is highly pathogenic to the upper respiratory tract and can cause severe kidney lesions. This study confirm a circulation of GI-23 strain in the country and report, to first time, the isolation of an exotic variant of IBV in Brazil.

## 1. Introduction

Avian infectious bronchitis (IB) is essentially a respiratory disease, but it can also affect the urinary system of young chickens. This disease in layer and breeder chickens may decrease the production and quality of eggs. Thus, IB causes significant economic losses to the poultry industry worldwide, and is one of the main respiratory diseases of chickens [1]. In Brazil, a country free of highly pathogenic viruses of Avian Influenza [2] and Newcastle Disease [3], IB represents one of the greatest health challenges in poultry farming.

Since 2018, the infectious bronchitis virus (IBV) has been designated as *Avian coronavirus*-ACoV [4]. It belongs to the order Nidovirales, family Coronaviridae, subfamily Orthocoronavirinae, and genus Gammacoronavirus. In addition to chickens, viruses from this genus infect other species, such as turkey, pheasant, and guinea fowl, in which it is also associated with disease [5]. ACoV is an epitheliotropic virus that affects epithelium-covered organs. The virus replicates primarily in the respiratory tract, mainly in the ciliated epithelium of the trachea and mucus-secreting cells [6]. It causes viremia for a short time, followed by systemic dissemination to other organs, in which further replication can occur, depending on virus strain and host immune status [7].

Due to the existence of various types of this virus, in 2016, a classification system based on the molecular sequence diversity of the S1 subunit was proposed (Valastro et al., 2016) [8]. This system classifies IBV strains into seven genotypes (GI to GVII). Some of these are well-established lineages such as GI-1, such as in Massachusetts (Mass), with a broad geographic distribution, which might be vaccine and vaccine-like strains [8]. In 1998, the first reported IBV case, a pathogenic variant Var-2 (genotype I lineage 23, GI-23), was detected in chickens suffering from respiratory and urinary problems in India [9]. This variant spread to other countries such as Jordan, Northern Iraq, Egypt (where it has circulated since 2010), Türkiye, Libya (respectively, in 2011 and 2012), and Nigeria, where the virus was detected for the first time in 2013 [10,11,12,13,14,15,16,17]. Afterward, it was detected in Saudi Arabia, Kuwait, Bahrain, and Armenia, and since 2015 this variant has also been isolated in Russia, Lithuania, Ukraine, Germany, and Poland [16,18,19]. Such evidence in several countries illustrates the ability of strain GI-23 to spread rapidly.

In Brazil, studies have also revealed the prevalence of IBV in chickens. Genotype I lineages 1 and 11 (GI-1 and GI-11) were the only variants identified until recently [20,21,22,23,24]. The high variability of serotypes and genetic genotypes that characterizes IBV worldwide does not occur in Brazil.

Inactivated and live-attenuated vaccines derived from Brazilian (BR) lineage 11 (GI-11) and Massachusetts (Mass) lineage 1 (GI-1) have been used to control IBV in Brazil. However, in some areas, small outbreaks continue to occur mainly due to intensive breeding systems [5]. In addition, intensive international trade, cross-border migration routes of wild birds, and uncontrolled movement of people and animals can pose an additional threat for the circulation and entry of new IBV variants in the country [16,17,18,19,20,21,22,23,24,25].

Recently, there has been an increase in cases of respiratory diseases in chicken-producing regions of Brazil (states of Paraná, Rio Grande do Sul, Santa Catarina, São Paulo, Goiás and Ceará). Through molecular diagnosis, the presence of the GI-23-type strain was detected in samples from poultry farms with different production performances and no other sanitary problem [26]. Consequently, there was a significant increase in slaughter condemnations. Therefore, samples from broilers with clinical signs were collected from poultry farms in the Paraná State for the detection and identification of IBV by RT–PCR, and characterization of the isolates by S1 gene sequencing. Furthermore, we report for the first time the pathogenicity of the isolate designated GI-23 in SPF birds (Specific Pathogen Free).

## 2. Materials and Methods

### 2.1. Clinical Disease

In 2021, there was an increase in case reports of broilers with clinical signs of mild respiratory disease and aerosaculitis lesions. In addition, a small increase in mortality was observed in the affected regions. The disease was recorded in areas of intensive poultry farms with different production performances and without other sanitary problems. One of the main problems of the outbreak was the high percentage of condemnations at the slaughterhouses, with peaks in June, July and August (winter). Some poultry houses could be more affected than others in the same farm.

Biological samples (419) were collected from broilers (14 to 41 days old) with respiratory symptoms from 11 poultry farms located in the South region, Paraná State, Brazil. The sampling was performed between August and December 2021 by veterinary technicians on each farm. The samples included tracheal swabs, lungs, kidneys, cecal tonsils, and oviducts. All samples were transported to the laboratory in insulated boxes with ice packs and stored at −70 °C until processing.

### 2.2. Viral Screening by Real-Time RT-PCR Assay

Initial screening was carried out to detect IBV in the collected biological samples.

A real-time RT-PCR assay was performed. A Taqman^®^-labeled probe was used to detect IBV in the collected biological samples by amplifying a 143-bp product in the 5′-UTR of the IBV genome [27] performed basically as follows: Sterile swabs with biological samples (tracheas, lungs, kidneys and cecal tonsils) were pooled and vortexed in 1 mL sterile phosphate-buffered saline (PBS) solution. RNA was detected from 200 µL of the homogenized sample by magnetic bead purification, using the IndiMag Pathogen Kit (Indical Bioscience, Leipzig, Germany) and following the manufacturer’s instructions. IBV-positive samples were classified into Mass (GI-1) or BR (GI-11) strain lineages [28]. Several samples of them were not classified as either GI-1 or GI-11. These samples were investigated for GI-23 genotypes through reverse transcription polymerase chain reaction (RT-PCR) followed by sequencing.

### 2.3. DNA Sequencing and Phylogenetic Analysis

RNA samples were subjected to cDNA synthesis with the High-Capacity cDNA Reverse Transcription Kit (Thermo Fisher Scientific, Cambridge, MA, USA) in agreement with the fabricator’s recommendations. Genome amplifications were performed using Spike S1 gene primers to obtain a complete or partial S1 sequence [16]. Sequencing was performed using ABI 3100 Genetic Analyzer (Applied Biosystems, Cambridge, MA, USA). Chromatograms were edited and aligned with the Clustal W through the Bioedit software [29]. Nucleotide sequences of the S1 gene from Brazil IBV strains obtained in this study were submitted to GenBank, subjected to BLAST (primary genotyping and similarity results), aligned, and compared with reference strains downloaded from the NCBI GenBank database. Sequence homology analysis and phylogenetic trees were constructed using MEGA11 with the Maximum Likelihood method (bootstrap values of 1000) and Tamura3-parameter model [30].

### 2.4. Virus Isolation and Titration in Specific Pathogen-Free Chicken Embryonated Eggs (SPF-CEE)

The first nine biological samples classified as GI-23 variants by molecular analysis were submitted to virus isolation. All samples were from broiler chicken farms of Parana State in the South of Brazil. Suspensions of tissues (10–20% *w*/*v*) were prepared in sterile phosphate-buffered saline (PBS), clarified by low-speed centrifugation and filtration through bacteriological filters (0.2 μM). Each sample was inoculated in SPF-CEE to virus isolation and incubated for five to seven days at 37 °C [31]. The viable virus was titred and expressed as 50% embryo infectious doses (EID_50_) according to the Reed and Muench (1938) [32]. Strains isolates were stored at the microbial culture collection (CMISEA) (Table 1) at Embrapa Swine and Poultry/Concordia/Brazil for pathotyping in vivo assays. The first three isolated strains were used for pathotyping assays.

### 2.5. Pathogenicity In Vivo Assays

#### 2.5.1. Experimental Design

Eight-day-old SPF chicks (*n* = 44 white leghorns) were used. Thirty-six chicks were assign to three groups with twelve birds each. The remaining eight chicks were kept as a negative control. All groups were housed in separate positive-pressure chambers. Three groups were inoculated with approximately 10^3.0^ EID_50_/bird (0.2 mL) of each IBV GI-23 isolate (BRMSA2916, BRMSA2917 and BRMSA2919) by intra-ocular and intranasal routes [33]. Clinical signs such as difficulty breathing, hunched posture, depression, emaciation, diarrhea and mortality were monitored from the first to the thirteenth-day post-infection (13 dpi). After 6 and 13 dpi, half of the birds in each group were euthanized. During the necropsy, pathological lesions were evaluated and tissue samples were collected. At 6 dpi, the tracheas were subjected to ciliary activity. At 6 and 13 dpi, portions of the tracheas, air sacs, lungs, and kidney fragments were collected for histopathological examination. Throughout the 13 days of the experimental period, birds received water and feed ad libitum and the room temperature was adapted according to the bird’s age.

#### 2.5.2. Ciliary Activity

Tracheas were removed from each chick and processed to determine ciliary activity according to scores previously described [34] with some modifications as follows: First, the ciliary activity was scored as zero (100 to 75% ciliary activity), 1 (<75 > 50% ciliary activity), 2 (<50 > 25% ciliary activity), and 3 (25 to 0% ciliary activity). Tracheas were sectioned into three portions (proximal, median, and distal), and three sections in each piece (1 to 2 mm) were examined by low-power microscopy, totaling nine rings/bird. It was considered that the mode from each portion and, finally, the mode of the three parts defined each bird’s final score of ciliary activity.

#### 2.5.3. Histopathological Examination

Fragments of the median trachea and kidney, plus fragments of air sacs and lungs, were placed in 10% buffered formalin, sectioned, stained with haematoxylin and eosin, and evaluated under optical microscopy [31].

The histological lesions were evaluated and classified in scores from greater to lesser severity according to conventional evaluation standards: grade zero when no significant lesion was observed, grade 1 for minor injuries, grade 2 for moderate injuries, and grade 3 when severe injuries were observed.

Morphological characteristics observed by histopathology in tracheas included the loss of cilia and epithelial cells, degeneration or necrosis of epithelial cells, degeneration of mucous glands, mucosal inflammatory infiltrates, and epithelial hyperplasia. Grades 2 or 3 were considered relevant lesions.

The scores of microscopic lesions in the kidney and the parameters evaluated included tubular degeneration and necrosis and the presence of inflammatory infiltrates.

### 2.6. Ethics Statement

The chickens were housed and handled at the Laboratory of Animal Health and Genetics Embrapa Swine and Poultry, Concordia- SC, Brazil. All procedures had been approved by the Embrapa Swine and Poultry Ethical Committee for Animal Experimentation (CEUA/CNPSA), in accordance with ethical principles and guidelines of animal experimentation adopted by the Brazilian College of Experimentation (process number: 01/2022; approval date: 3 October 2022).

## 3. Results

### 3.1. IBV Screening by RT-qPCR and Sequencing of GI-23 Lineage

**Figure 1:**
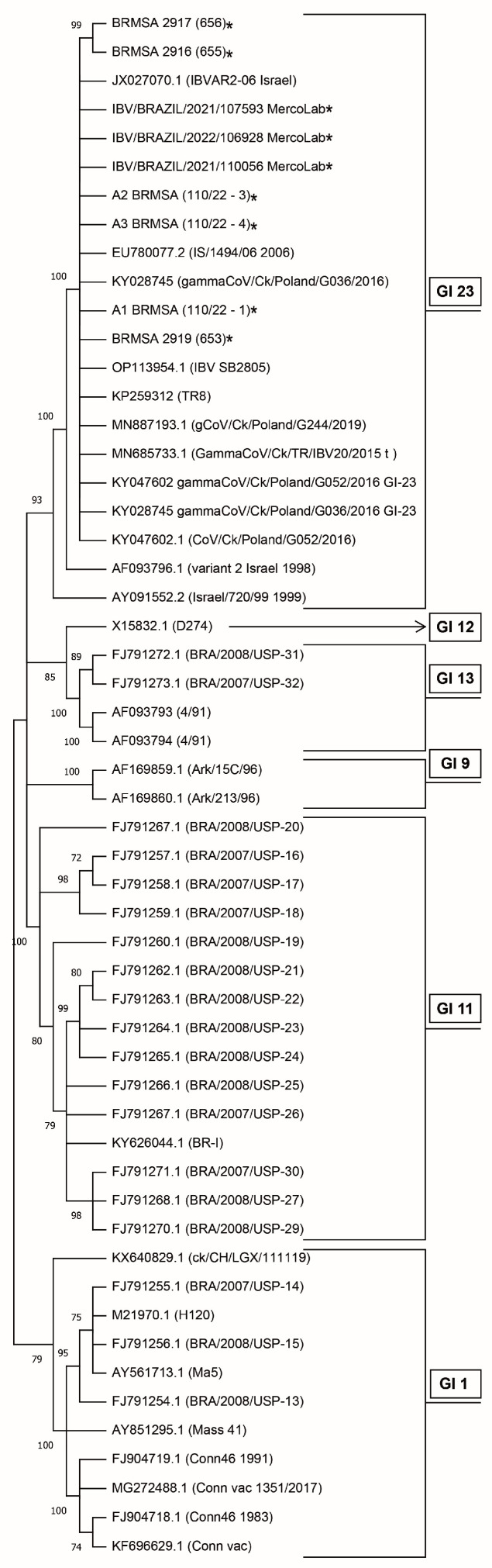
Phylogenetic relationships based on partial spike gene nucleotide sequences of avian infectious bronchitis virus (IBV) detected in Brazil. The inferred evolutionary history using the Maximum Likelihood method and Tamura 3-parameter model [35]. It showed the tree with the highest log likelihood (−4060.44). The percentage of trees in which the associated taxa clustered is shown next to the branches. The initial tree(s) for the heuristic search were obtained automatically by applying Neighbor-Join and BioNJ algorithms to a matrix of pairwise distances estimated using the Tamura 3 parameter model and then selecting the topology with superior log likelihood value. The rate variation model allowed some sites to be evolutionarily invariable ([+I], 45.19% sites). This analysis involved 54 nucleotide sequences. There were a total of 1807 positions in the final dataset. MEGA11 was used for evolutionary analyses [30]. The Genbank sequence I.D. OP113954.1 (IBV SB2805), was the first IBV sequence that showed a pattern similar to other sequences of the GI-23 group in Brazil [26]. * Indicate the sequences obtained from the samples isolated and analyzed in the present study.

Overall, 419 biological samples from chickens with suspected IB were subjected to analyzes. According to RT-PCR screening, 224 (53.46%) of them were positive for IBV. From such positive samples, 47.77% (107) were not classified into genotypes GI-1 (Mass strains) or GI-11 (Brazilian autochthonous strains) frequently detected in Brazil. Among the group assigned as “not classified”, nine isolates were subjected to S1 glycoprotein gene sequencing. In the phylogenetic analysis, we used a sequence dataset of the strains frequently found in Brazil and GI-23 [26]. According to the phylogenic relationship shown in Figure 1, all nine isolates were grouped within the GI-23 strain. For three isolates, the analysis was based on a partial sequence of S1 with 308 nucleotides (NT) and 103 amino acids (AA) (GenBank IDs: ON470440.1; ON470441.1 and ON470442.1). The other six isolates had complete S1 sequencing (GenBank IDs: OQ573556; OQ573557; OQ573558; OQ573559; OQ573560; OQ573561-position 1 to 1804 NCT/1 to 601 AA).

### 3.2. Virus Isolation in SPF CEE

**Figure 2:**
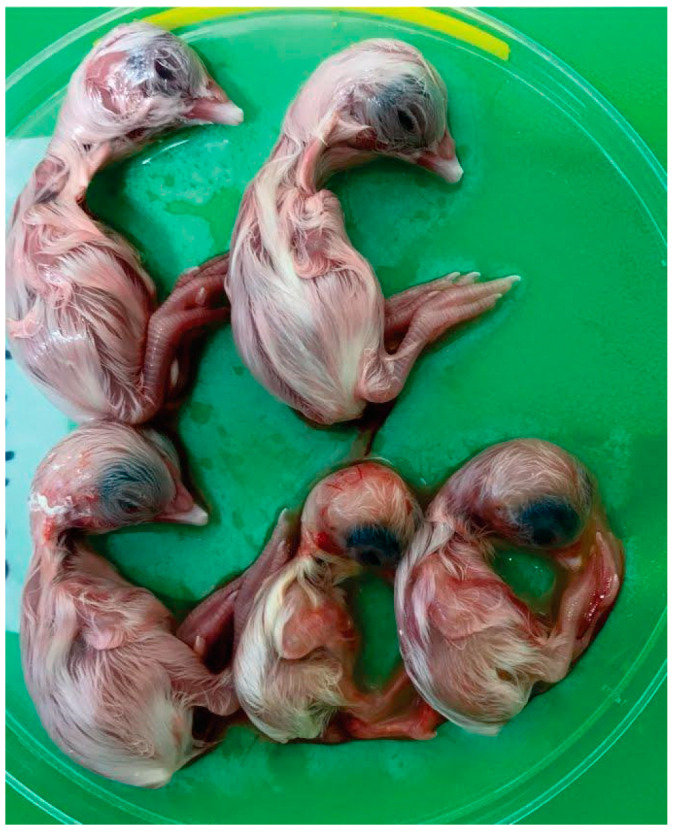
Embryo development at 17 days following inoculations with IBV GI-23 Brazilian field. Note evidence of dwarfing, winding, and hyperemia in the three IBV-infected embryos (bottom of figure) compared to a non-infected control embryo (two larger embryos at the top).

Six GI-23 strains of IBV were successfully isolated from biological samples after four to five passages in SPF CEE (Table 1). According to Figure 2, infected embryos showed typically IBV-induced changes such as dwarfism, curling, and hyperemia. Subsequently, the presence of the virus was confirmed by RT-PCR in the chorioallantoid fluid of the eggs. The titers of the isolates were 10^6.3^ EID_50_/200 uL, 10^4.3^ EID_50_/200 uL and 10^3.6^ EID_50_/200 uL, respectively, for BRMSA2916, 2917 e 2919.

### 3.3. Pathogenicity In Vivo Assays

#### 3.3.1. Clinical Signs and Gross Pathology

The strains BRMSA2917, BRMSA2919, and BRMSA2916 were evaluated “in vivo” regarding their virulence abilities. During the experimental period, clinical signs such as difficulty breathing, hunched posture, depression, emaciation, diarrhea and mortality, were not observed in the chicks inoculated with viruses. The evaluation did not consider other respiratory signs, such as tracheal rales, sneezing and coughing, since keeping the chicks in positive-pressure isolation chambers made it difficult to assess these parameters. No clinical signs or mortality were observed in the control group.

**Figure 3:**
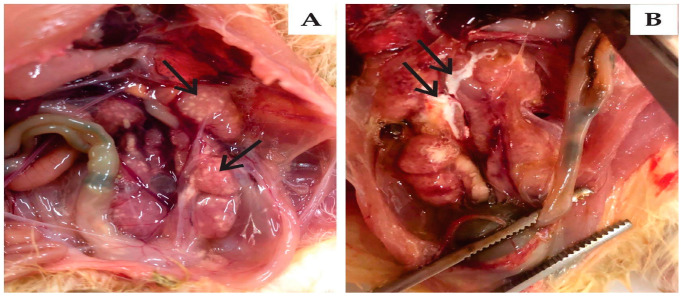
Gross pathology in kidney. The arrows in (**A**) indicate the enlarged kidney lobes and in (**B**) they indicate the presence of urates in kidney lobes.

**Figure 4:**
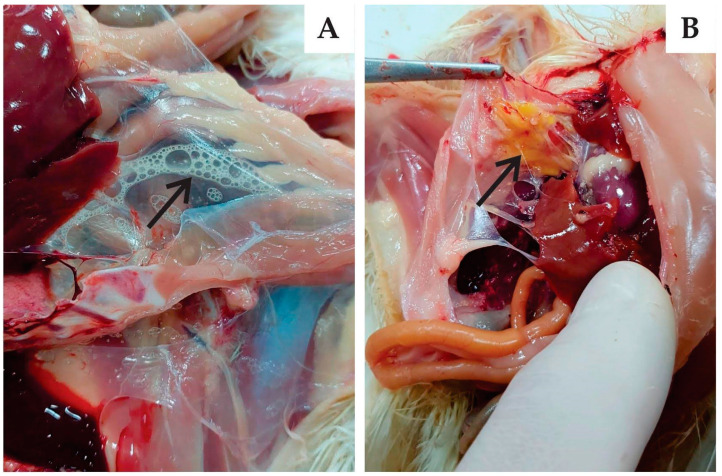
Gross pathology in air sacs infected with Brazilian GI-23 IBV. The arrow in (**A**) indicate the presence of foam and opacity at 6 dpi, and the arrow in (**B**) shows the presence of yellowish material in the air sac 13 dpi.

For all three strains, the main observation at 6-dpi necropsy was the presence of mucus in the tracheas and air sac opacity in some of infected birds. The main injuries were observed in the kidneys, including enlarged organ size and the presence of urates (Figure 3). These injuries occurred in 17% of birds infected with BRMSA2917(656), 50% of those with BRMSA2919(653), and 67% of those with BRMSA2916(655). At 13 dpi, for all groups, no lesions were observed in the lungs or kidneys. An interesting pathological finding was the presence of foam and opacity in the abdominal air sacs in chickens of all inoculated groups (Figure 4). However, the incidence was higher in the BRMSA2917(656) group (50%) compared to the others.

#### 3.3.2. Ciliary Activity

According to ciliary activity (CA) data in Table 2, all birds within inoculated groups were affected by ciliostasis at 6 dpi. At this same age, GI-23 IBV strains showed marked ciliostasis (score 3) on tracheas. At 13 dpi, all birds in the group infected with GI-23 BRMSA2919 continued to show ciliostasis. On the other hand, the ciliostasis incidence in groups infected with IBV GI-23 BRMSA2916 and BRMSA2917 both decreased to 67%.

#### 3.3.3. Histopathological Examination

**Figure 5:**
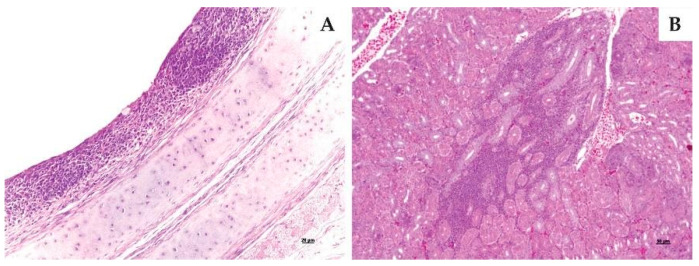
Histopathology of tracheal mucosa and kidney. (**A**) indicates the tracheal mucosa with lymphoplasmacytic infiltration, epithelial cell hyperplasia, ciliary loss and degeneration of epithelial cells; (**B**) indicates kidney interstitial lymphoplasmacytic infiltration.

At 6 dpi, the main microscopy changes had been in the tracheas. The lesions were present in 67%, 83% and 100% of birds, respectively to BRMSA2916, BRMSA2917, and BRMSA2919 strains. The pathological changed were characterized by lymphoplasmacytic inflammation in the mucosa, ciliary loss in the mucosal epithelial cells, and epithelial hyperplasia. At 13 dpi, the lesions were the same, although to a lesser extent for the three strains. All lesions are compatible with IBV (Table 2 and Figure 5A).

Regarding the kidneys at 6 dpi, the development of lesions was observed in 83% of chickens for BRMSA2916, 50% for BRMSA2917, and 17% for BRMSA2919 (Table 2 and Figure 5B). At 13 dpi, all strains induced similarly significant lesions in one to two birds. On lung evaluation, the injuries were inconsistent, occurring only in some birds.

Microscopic lesions were not identified in the air sacs at 6 dpi. On the other hand, at 13 dpi, it was possible to observe thickening due to inflammatory infiltration, with a predominance of heterophils, hyperemia and mild epithelial hyperplasia in many birds (4/6) forthe BRMSA2917 and BRMSA2919 strains.

## 4. Discussion

The main issue regarding IB is the frequent appearance of new variant strains of IBV by mutation or recombination. This fact generates a concern, since the available vaccines may have limited efficacy against the emergence of these new strains. The IBV variant GI-23 was reported as causing disease in birds for the first time in India in 1998. After this, it spread rapidly across different continents. In Brazil, the variability of IBV strains is considered limited, probably as a consequence of low pressure by unique live vaccine serotypes in contrast to that previously described, in which the introduction of several new vaccine serotypes probably affected the speed of evolution of IBV in the field [33]. In addition to vaccine effectiveness, proper management and biosecurity can reduce the spread and diversification of IBV serotypes and genotypes. Recently, however, this scenario changed. Since early 2021, some intensive broiler-producing areas have noticed an increase in respiratory disease with severe airsacculitis and great economic losses at slaughterhouses.

To investigate health causes, four hundred and nineteen (419) biological samples were analyzed. Firstly, we ran diagnostic tests to discharge other infectious agents that could cause similar respiratory signs. After negative analysis for agents such as mycoplasmas, metapneumovirus, and laryngotracheitis virus, we identified 224 samples (53%) in which IBV occurred as the sole respiratory pathogen. In the initial screening, we found different well-defined groups of IBV coexisting nowadays in Brazil: GI-1 (Massachusetts), GI-11 (Brazilian autochthonous strains), and 107 (48%) of them were not confirmed for the Massachusetts- or Brazilian-strain-lineage GI-11 (BR-I and BR-II). Unclassified variants in the GI-1 and GI-11 lineages were sequenced and the phylogenetic analysis (Figure 1) showed new viral strains, classified as GI-23. This result is in agreement with earlier research reported by Ikuta et al. (2022) [26]. Although, in general, the amino acid sequences obtained in the present study were very similar to the sequences presented by Ikuta et al. (2022) [35] (Table 1 of such work), through the analyses carried out in the present study, some differences were found. These differences could be related to genetic variations linked to the area where the viruses were collected.

In this context, the lack of knowledge about the virulence of these new strains is a limiting factor for disease control. Therefore, in this study, we demonstrate for the first time the pathogenicity of the novel strain identified as IBV GI-23 in SPF chickens in Brazil.

During the in vivo assay, clinical signs such as respiratory distress, depression, diarrhea or mortality were not observed. Zanaty et al. (2016) [36] observed severe respiratory signs and mortality of up to 50% in day-old SPF chicks inoculated with IBV GI-23 from Egypt; while Lisowska et al. (2021) [37] observed respiratory symptoms, ruffled feathers, depression and 30% mortality in day-old chicks inoculated with IBV GI-23 strains from Poland. On the other hand, Awad et al. (2016) [38] reported mild tracheal rales, sneezing, coughing and only 2.2% mortality, in day-old SPF chicks. Such findings were similar to that observed with the strains from Brazil, in 8-day-old chicks [38]. Many factors can contribute to the differences found in the in vivo studies, including the viral infectious dose, the age and lineage of the birds, the virulence associated with the geographic evolution of the each strain, route of inoculation, among others [6]. In the studies cited here, the challenge dose was significantly different. To define the pathotypes of IBV GI-23, the titers were adjusted to 10^3.0^ EID_50_/bird, according to OIE, 2013 (validation of live IBV vaccines) [39]. The immunization dose of the current vaccination programs used in Brazil for IB is approximately 10^3.0^ EID_50_/bird. In the ongoing protectotype assays, the same dose of challenge and vaccination is always used. We understand that the same dose is adequate to determine the efficacy of vaccines and that higher challenge doses would not represent the reality of the field.

The infectious dose was established as 10^5.00^ EID_50_/bird by Zanaty et al. (2016) [36] and Lisowska et al. (2021) [37], while in the present study and in the study of Awad et al. (2016) [38], the doses applied were 10^3.66^ EID_50_/bird and 10^3.00^ EID_50_/bird, respectively. Zanaty et al. (2016) [36] concluded that the most severe clinical symptoms of IBV appear in very young chicks and the severity decreases in older chickens. Lisowska et al. (2021) [37] agree with that, summarizing that the pathogenicity of GI-23 is more severe in young birds. To Awad et al. (2016) [38], the disease was of a lesser severity in broilers compared to SPF chicks, reflecting the inhibitory effects of the IBV maternal antibodies or genetic/strain susceptibility, or both. All this emphasized that the virulence of IBV strains in birds depends on many factors.

The main macroscopic lesions of the trachea caused by the Brazilian GI-23 IBV variants were composed of mucus and congestion, corroborating with Awad et al. (2016) [38] and Lisowska et al. (2021) [37], who observed severe congestion in the trachea and lungs.

IBV GI-23 strains BRMSA2916, BRMSA2917, and BRMSA2919 induced similar and significant tracheal microscopy lesions (lymphoplasmacytic inflammation, ciliary loss in the mucosal epithelial cells, and epithelial hyperplasia), all compatible with IBV infection. Awad et al. (2016) [38] described similar microscopy lesions (loss of cilia and heterophil infiltration, decreased mucous cells and an occasional heterophilic exudate in the tracheal lumen) and Zanaty et al. (2016) [36] related deciliation, degenerative changes and edema of the tracheal mucosa. Infectious bronchitis starts essentially through the airways, regardless of the tissue tropism of the strains; therefore, tracheal lesions will always be seen in pathogenic strains.

Birds in the groups inoculated with BRMSA2916 and BRMSA2917 showed recovery in ciliary activity from 0% at 6 dpi to 33% at 13 dpi. This result corroborates studies on the pathogenicity of the GI-11 (BR) strains. On the other hand, birds in the group challenged with BRMSA2919 maintained total ciliostasis at 6 and 13 dpi (Table 2), suggesting that this strain is more virulent for the upper respiratory tract.

The presence of foam and opacity in the abdominal air sacs at 6 dpi and at 13 dpi, evolving to yellowish material (Figure 4), although present in a few birds/group, occurred in birds of all evaluated strains to mild/moderate degrees. These samples showed microscopic lesions not compatible with bacterial infection. Butcher et al. (1990) [40] reported a thickening and edema of the air sacs in broiler chicks challenged with a nephrophatogenic strain and Jackwood and de Wit (2020) [6] reported foamy airsacculitis associated with the Delaware 072 strain. Lisowska et al. (2017, 2021) [16,37] described very evident and common lesions in the air sacs of chicks infected with GI-23 IBV from Poland. We have never reproduced air sac lesions in the experimental challenges with GI-11 strains at the similar conditions. A larger number of samples are evaluated for the presence of IBV and bacterial agents.

Lesions observed in the lungs occurred inconsistently, being present in only a few birds and, to a mild degree, agreeing with Lisowska et al. (2021) [37]. Our trials have shown that IBV rarely causes lung damage.

The sample IBV-BRMSA2916 (655) induced significant interstitial inflammatory kidney lesions, demonstrating a nephropathogenic condition, mainly at 6 dpi. This finding is in agreement with Awad et al. (2016) [38], Zanaty et al. (2016) [36] and Lisowska et al. (2021) [37], that concluded that GI-23 IBV strains have the strongest tropism to the renal tract. At 13 dpi, interestingly, none of the birds showed visible kidney pathology. Lesions were expected to be more severe at 13 dpi than at 6 dpi. Among the three Brazilian samples studied, only one induced kidney damage. The complete genome sequence of the viruses may provide information on differences in the pathogenicity of the strains, as well as some indication of the source of the GI-23 variant in Brazilian poultry production.

## 5. Conclusions

Our findings indicated that GI-23 strains of IBV (specifically the variants BRMSA2916, BRMSA2917, BRMSA2919) exhibit high pathogenicity to embryos and to the respiratory tract of SPF chicks. We observed that this respiratory disease can cause the curling, dwarfism and hyperemia in the embryos; aerosaculitis; presence of foam and opacity in the abdominal air sacs; lymphoplasmacytic inflammation in mucosal; increased kidney size; kidney damage with the presence of urates; and ciliostasis. In addition, this study confirmed the presence of IBV GI-23 lineage circulating within Brazil. Such evidence suggested that GI-23 strains of IBV may be involved in clinical respiratory disease of chickens in Brazil. This is the first report of isolation of an exotic variant of IBV in Brazil.

## Figures and Tables

**Table 1:** Brazilian field strains from the South of Brazil, Parana State classified as GI-23 variants by molecular analysis.

Virus/Strain *	Age (Days)	Organ Isolated	Symptom	Year of Isolation
IBV-BRMSA2916 (655)	23	Cecal tonsil	Respiratory	2021
IBV-BRMSA2917 (656)	41	Trachea	Respiratory	2021
IBV-BRMSA2919 (653)	23	Cecal tonsil	Respiratory	2021
IBV-BRMSA3090 (110-3)	27	Kidney	Respiratory	2022
IBV-BRMSA3091 (110-4)	27	Kidney	Respiratory	2022
IBV-BRMSA3092 (110-1)	15	Lung	Respiratory	2022

* BRMSA: Brazil Microorganisms Swine and Poultry and numbers in parentheses are the laboratory codes.

**Table 2:** Ciliostasis and microscopy lesions of tracheas and kidney at 6 and 13 dpi.

Group	Tissues	Score and Percentage/Group *									
		6 dpi					13 dpi				
		0	1	2	3	%	0	1	2	3	%
Negative Control (NC)	CA	4	0	0	0	0	4	0	0	0	0
	T	4	0	0	0	0	4	0	0	0	0
	K	4	0	0	0	0	4	0	0	0	0
BRMSA2916 (655)	CA	0	0	0	6	100	0	2	2	2	67
	T	0	2	4	0	67	0	2	3	0	60
	K	0	1	2	3	83	1	3	2	0	33
BRMSA2917 (656)	CA	0	0	0	6	100	0	2	2	2	67
	T	0	1	3	2	83	0	2	2	1	50
	K	3	0	2	1	50	2	2	1	1	33
BRMSA2919 (653)	CA	0	0	0	6	100	0	0	3	3	100
	T	0	0	2	4	100	0	2	3	1	67
	K	3	2	0	1	17	4	2	1	0	17

* Number of birds in each score. (CA) Ciliary Activity, (T) Trachea, (K) Kidney, (**%**) Ciliostasis (scores ≥ 2).

## Data Availability

The details data supporting reported results was in kept in lab diary and can be found, in our Research Institution.
